# Unmasking Arrhythmia Mortality: A 25‐Year Analysis of Trends and Disparities in the United States (1999–2023)

**DOI:** 10.1002/clc.70109

**Published:** 2025-03-04

**Authors:** Bazil Azeem, Laiba Khurram, Bakhtawar Sharaf, Arwa Khan, Ayesha Habiba, Rabia Asim, Muskan Khelani, Hamza Ali, Abdul Hadi Ansari, Tazheen Saleh Muhammad, Muhammad Abdullah Naveed, Mata‐e‐Alla Dogar, Aalaa Saleh, Hamza Ashraf

**Affiliations:** ^1^ Department of Internal Medicine Shaheed Mohtarma Benazir Bhutto Medical College Lyari Karachi Pakistan; ^2^ Department of Internal Medicine Dow Medical College, Dow University of Health Sciences Karachi Pakistan; ^3^ Faculty of Medicine Lebanese University Beirut Lebanon; ^4^ Department of Medicine Allama Iqbal Medical College Lahore Pakistan

**Keywords:** arrhythmi, CDC WONDER, mortality, USA

## Abstract

**Background:**

Arrhythmias are a significant cause of cardiovascular mortality in the U.S. This study examines trends in arrhythmia‐related mortality from 1999 to 2023, focusing on gender, racial, regional disparities, and specific arrhythmic conditions.

**Objective:**

To analyze trends and disparities in arrhythmia‐related mortality among U.S. adults aged ≥ 35 years from 1999 to 2023, with a focus on the impact of sex, race, geographic location, and urbanization.

**Methods:**

We analyzed mortality data from the CDC WONDER database, focusing on deaths where arrhythmias were a contributing cause. Age‐adjusted mortality rates (AAMRs) were calculated and stratified by sex, race/ethnicity, state, and region. The annual percentage change (APC) and average annual percentage change (AAPC) were estimated using Joinpoint regression.

**Results:**

A total of 5,050,271 arrhythmia‐related deaths were recorded, with the overall AAMR increasing from 111.4 in 1999 to 137.3 in 2023. Mortality rates declined significantly from 1999 to 2009 (APC: −1.04%; *p* = 0.003) but rose sharply from 2009 to 2018 (APC: 1.69%; *p* = 0.003), peaking in 2021 during the COVID‐19 pandemic (APC: 8.63%; *p* < 0.001). A subsequent decline was observed from 2021 to 2023 (APC: −3.91%; *p* = 0.044). Males consistently exhibited higher AAMRs than females (137.2 vs. 95.3), as did non‐Hispanic White individuals compared to other racial groups. Geographic disparities revealed higher mortality rates in Nonmetropolitan areas and the Midwest, with the highest AAMR observed in Oregon and the lowest in Hawaii.

**Conclusion:**

Despite an overall decline in arrhythmia‐related mortality, recent increases, especially in West Virginia and among certain racial groups, highlight the need for targeted public health interventions.

## Graphical abstract

A 24‐Year Analysis of Trends and Disparities in Arrhythmia‐Related Mortality in the USA.

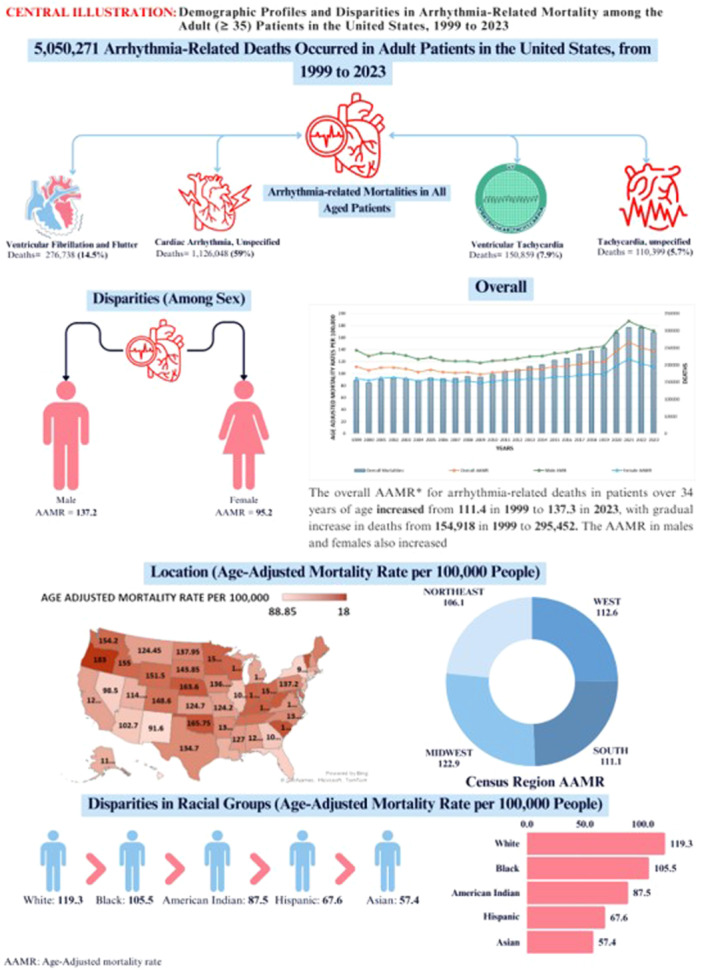

## 1 Introduction

Cardiovascular disease (CVD) remains the leading cause of disability and death in the United States, responsible for 1 in every four deaths [1]. Among CVDs, arrhythmia is a major contributor to mortality and morbidity, affecting millions of adults each year [2]. Arrhythmias, characterized by irregular heartbeats, encompass various conditions, including atrial fibrillation (AF), ventricular fibrillation, bradyarrhythmias, and premature depolarizations [3]. These cardiac rhythm disorders can significantly impact patient outcomes, leading to increased rates of disability, sudden cardiac death, and long‐term complications [4].

The prevalence of arrhythmias has been rising in the United States, driven by an aging population and the growing burden of risk factors such as hypertension, diabetes, and obesity [5]. Arrhythmia‐related death rates have shown an upward trend over the past two decades. Notably, from 1999 to 2017 [6], mortality rates associated with arrhythmias increased, with significant spikes observed in ventricular arrhythmias such as ventricular fibrillation and flutter [7]. In addition, the COVID‐19 pandemic exacerbated arrhythmia‐related mortality, with marked increases in deaths during the years 2020–2022, potentially due to both direct and indirect effects of the pandemic on cardiovascular health [8].

Although these trends in arrhythmia‐related mortality have been well‐documented, existing studies often aggregate adult populations without focusing on specific age groups. Research has predominantly examined arrhythmia mortality across all adults, but few studies have investigated how these trends differ in adults. Given the increasing burden of arrhythmia‐related morbidity and mortality in this age group, understanding age‐specific mortality patterns is essential for informing public health strategies and improving patient care. Therefore, this study aims to analyze trends in arrhythmia‐related mortality among U.S. adults aged 35 and older from 1999 to 2019, emphasizing demographic differences and the influence of the COVID‐19 pandemic.

## 2 Methods

### 2.1 Population and Study Setting

We used the CDC WONDER database to collect information from death certificates. This study, covering the years 1999 to 2023, analyzed arrhythmia‐related deaths. The International Classification of Diseases and Related Health Problems, 10th edition (ICD‐10), diagnostic codes I44.0(I44.1, I44.2, I44.3, I44.4, I44.5, I44.6, I44.7), I45.0(I45.1, I45.2, I45.3, I45.4, I45.5, I45.6, I45.8, I45.9), I47.0(I47.1, I47.2, I47.9), I48.0, and I49.0(I49.1, I49.2, I49.3, I49.4, I49.5, I49.8, I49.9) were utilized in the study [9]. These codes or conditions are referred to as arrhythmia by previous studies [10, 11]. The data includes mortality records from all fifty states and the District of Columbia, detailing the specific causes of death listed on the certificates. Patients in this study were defined as individuals aged 35 to more than 85 years at the time of their death. In addition, sensitivity analysis as the underlying cause of death in the background of arrhythmia‐related cardiovascular disease was also carried out by using code (I00‐I99). No regional institutional review board approval was required since the study used government‐issued deidentified public use data. This study complied with STROBE guidelines for reporting observational research.

### 2.2 Data Abstraction

The data we gathered includes population size, year, place of death, demographics, geographic division, state‐specific data, and urban and rural classifications. Places of death include medical care facilities, hospices, nursing homes/long‐term care, and decedent's homes. Demographics cover age, sex, and race or ethnicity. Race is further categorized into non‐Hispanic (NH) White people, NH Black or African American people, NH American Indians or Alaska Native people, NH Asian or Pacific Island people, and Hispanic or Latino people. This data, which relies on reported death certificates, has also been used in previous research utilizing the WONDER database [9] The National Center for Health Statistics Urban‐Rural Classification Scheme categorizes the population into urban and rural areas. Urban areas include medium/small and large metropolitan areas, while rural regions have smaller populations [12]. This classification is based on the 2013 U.S. Census. Additionally, the United States Census Bureau's criteria were used to geographically divide areas into the Northeast, Midwest, South, and West regions.

### 2.3 Statistical Analyses

We calculated mortality rates per 100,000 individuals using age‐adjusted data from 1999 to 2023 to explore regional differences in arrhythmia‐related mortality. These rates were organized into distinct groups based on year, gender, race/ethnicity, state, and whether the area was urban or rural. For each group, we provided 95% confidence intervals (CIs). To find the crude mortality rates, we divided the number of arrhythmia‐related deaths by the corresponding U.S. population each year. The age‐adjusted mortality rates (AAMR) were then calculated by standardizing the arrhythmia‐related deaths in the U.S. population in 2000 [13]. This study aimed to investigate yearly variations in mortality from arrhythmia. To analyze annual trends in mortality due to arrhythmia at the national level, the annual percent change (APC) and its corresponding 95% confidence interval (CI) in AAMR were calculated using the Joinpoint Regression Programme (Version 5.0.2, National Cancer Institute) [14]. A significance level of *p* < 0.05 is set to determine statistical significance. A subgroup analysis is performed separately to find which types of arrhythmia are the leading cause of death; for simplicity and broader understanding, no restriction of the age group for this analysis has been applied, and a total number of deaths over the study period has been calculated. Those subtypes with at least 5% of mortalities are selected for analysis, while others are pooled together in other arrhythmias.

## 3 Results

A total of 5,050,271 arrhythmia‐related deaths occurred in the adults of age group of ≥ 35 years from 1999 to 2023 (Supplemental Table 1), among which information regarding place of death was available only for 4,852,772 of total deaths in which 49% occurred at medical facility, 23.05% took place in nursing homes/health care facilities, 3.75% in hospice facility while 24.56% happened at home (Table S2).

### 3.1 Annual Trends for Arrhythmia‐Related Mortality

**Figure 1:**
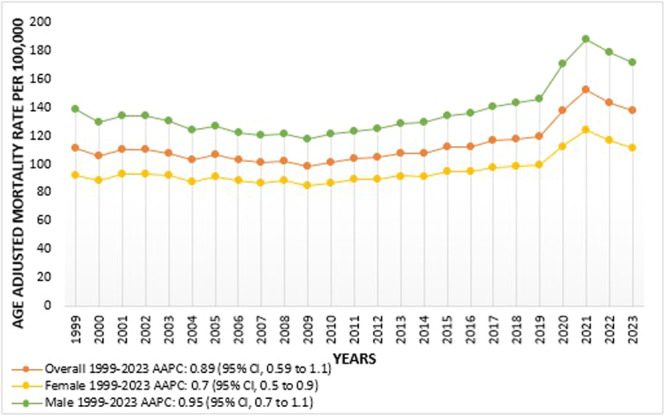
Overall and Sex‐Stratified Arrhythmia‐related AAMRs per 100,000 Among the population of the United States, 1999 to 2023.

AAMR for arrhythmia‐related deaths was 111.4 in 1999, and it jumped to 137.3 in 2023. A visible decline was seen in the overall AAMR from the year 1999 to 2009 (APC: −1.0433; 95% CI: −2.4256 to 0.4083; *p*‐value: 0.003199) however, a reversal in trend was observed from 2009 to 2018 (APC: 1.6909; 95% CI: 0.8471 to 2.5505; *p*‐value: 0.003199) trend reached its peak in 2021 (APC: 8.6275; 95% CI: 6.0919 to 9.9788; *p*‐value: 0.000400) followed by a significant reduction in overall AAMR from 2021 to 2023 (APC: −3.9134; 95% CI: −7.5544 to −0.1643; *p*‐value: 0.043991) (Figure 1; Tables S3 and S4; Figure S1). However, on sensitivity analysis, overall AAMR was estimated at 81.30 for 1999 and 73.50 for 2023; the trend shows a substantial decrease in AAMR from the year 1999 to 2010 (APC: 2.4738; 95% CI: −1.9662 to −3.1492; *p*‐value: < 0.000001) following which there was a rise in mortality from the year 2010 to 2023 (APC: 1.5310; 95% CI: 2.0886 to 1.1190; *p*‐value: < 0.000001) (Figure S2A,S2B).

### 3.2 Trends for Arrhythmia‐Related Mortality Stratified by Sex

**Figure 2:**
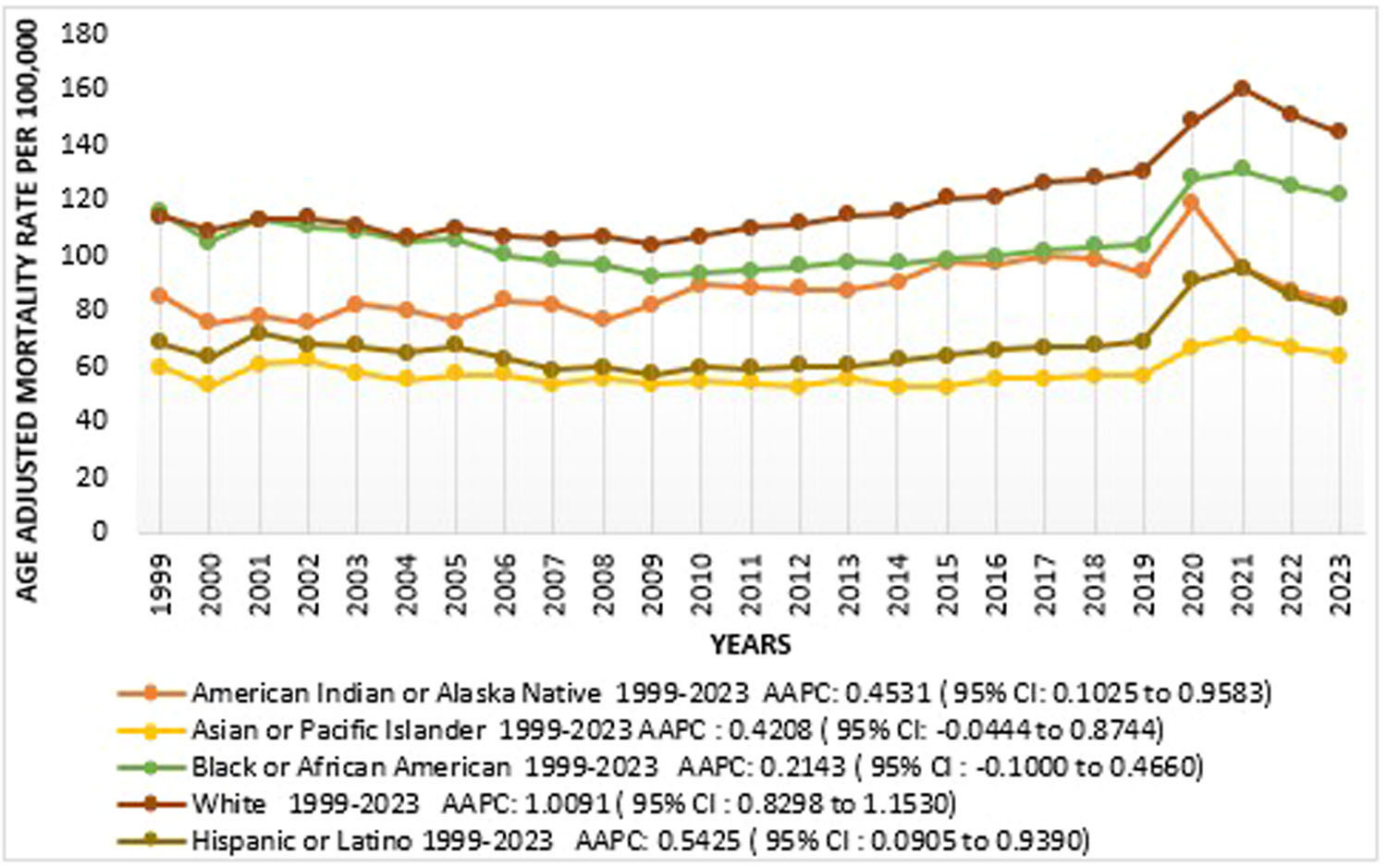
Arrhythmia‐related AAMRs per 100,000 Stratified by Race in the United States, 1999 to 2023.

AAMR stratified according to sex shows the overall value of AAMR in males was higher than in females between 1999 and 2023 [(Overall AAMR for males: 137.208; 95% CI: 136.32 to 138.08), (females overall AAMR: 95.268; 95% CI: 94.668 to 95.868)]. Beginning with 1999, males of the age group ≥ 35 years have an AAMR of 138.80. Trends show rapid decline in mortality rate from 1999 to 2009 (APC: −1.4406; 95% CI: −2.3925 to −0.8766; *p*‐value: < 0.000001) followed by increment in death rates up to 2018 (APC: 1.9540; 95% CI: 1.0960 to 2.7309; *p*‐value: < 0.000001) which reached its peak on 2021 (APC: 9.6080; 95% CI: 7.2747 to 11.0176; *p*‐value: < 0.000001) then by more steeper decline in trend between 2021 and 2023 (APC: −3.7450; 95% CI: −6.4373 to −1.0941; *p*‐value: 0.016397). Similarly, in the year 1999, females had AAMR of 92.10 and of 111.40 in 2023, with a slight but significant decrease in mortality trend between 1999 and 2009 (APC: −0.6905; 95% CI: −1.8858 to −0.2106; *p*‐value: 0.004399) following which trends show a rise to 2018 (APC: 1.2617; 95% CI: 0.4588 to 2.2346; *p*‐value: 0.001200) up surging to its peak in 2021 (APC: 8.1276; 95% CI: 5.9654 to 9.4630; *p*‐value: < 0.000001) followed by drop in trend from 2021 to 2023 (APC: −4.5950; 95% CI: −7.1562 to −2.1394; *p*‐value: < 0.000001) (Figure 2, Table S3 and S4; Figure S1).

### 3.3 Trends for Arrhythmia‐Related Mortality Stratified by Race/Ethnicity

**Figure 3:**
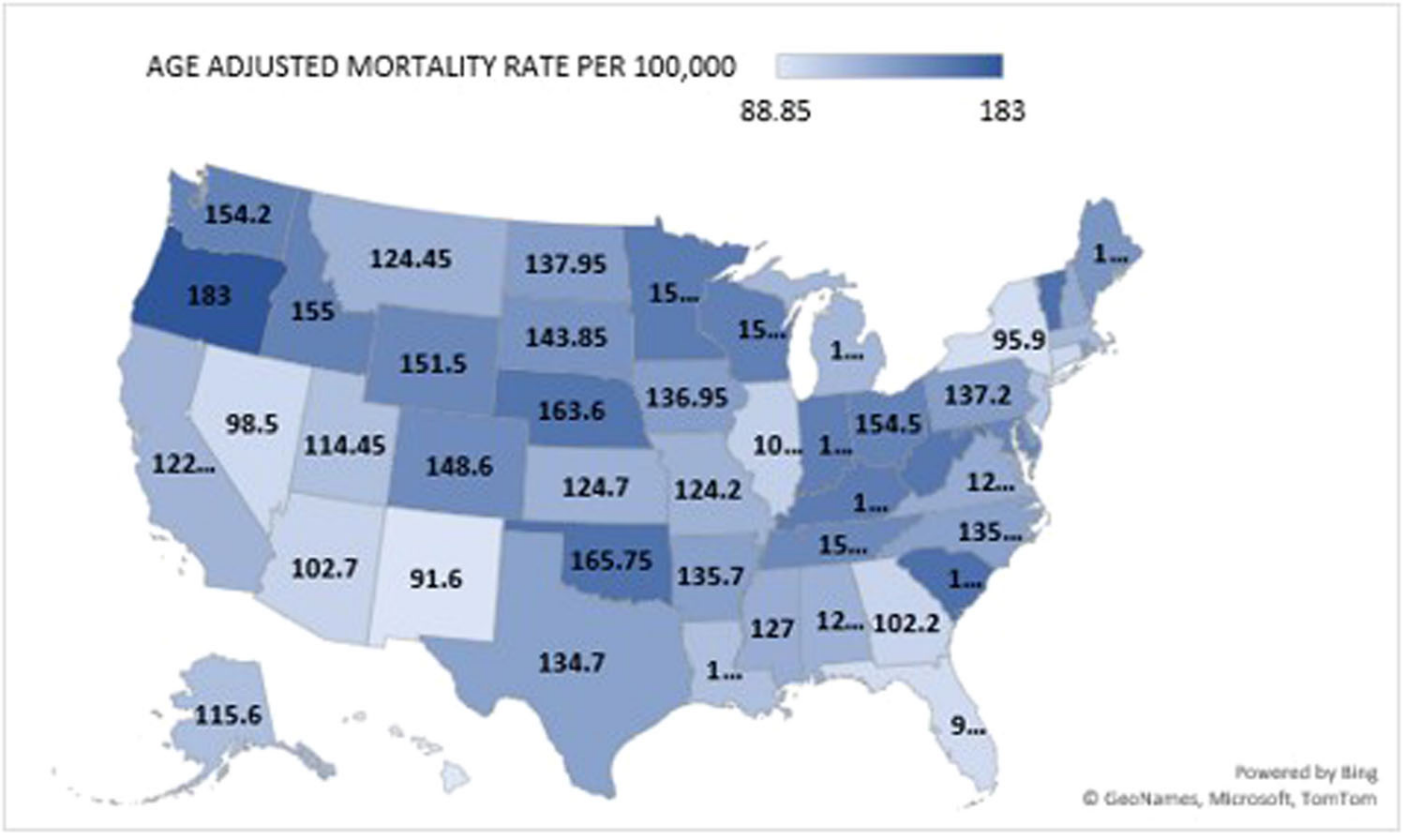
Arrhythmia‐Related AAMRs per 100,000 Stratified by State Among Adults in the United States, 1999 to 2023.

Disparities in AAMR are also present when stratified by race, i.e. Highest value for AAMR for 1999 to 2023 was estimated for Whites NH followed by Black or African Americans, American Indian or Alaska Native, Hispanics or Latino, Asian or Other Pacific Islander[(NH Whites has overall AAMR: 119.36; 95% CI: 118.788 to 119.936), (AAMR value for NH Black or African American: 105.52; 95% CI: 103.852 to 107.172), (NH American or Alaska Native AAMR: 87.532; 95% CI: 81.044 to 94.048), (Hispanics or Latino AAMR: 67.648; 95% CI: 66.084 to 69.204), (Asian or Other Pacific Islander AAMR: 57.42; 95% CI: 55.424 to 59.424)]. Notable trends were seen in NH Whites, showing a gradual but significant decline in death rates between 1999 and 2009 (APC: −0.7697; 95% CI: −1.4209 to −0.3425; *p*‐value: 0.000400) followed by a rapid surge in mortality rates as evident by increasing trend between 2009 and 2018 (APC: 2.1049; 95% CI: 1.3663 to 2.7272; *p*‐value: < 0.000001) and in 2021 there was a more prominent rise in AAMR reaching its peak value (APC: 7.9085; 95% CI: 6.0833 to 9.0395; *p*‐value: < 0.000001) then there is a recent steeper drop in death rates in 2021 to 2023 (APC: −4.7619; 95% CI: −6.9044 to −2.7812; *p*‐value: < 0.000001). Similar trends were seen in Hispanics and Black or African Americans; from 1999 to 2010, there was a visible decrease in death rates, however more steeper in Black than Hispanics[(APC value for Black or African Americans: −1.8583; 95% CI: −3.3107 to −1.2364; *p*‐value: 0.004399), (APC value for Hispanics: −1.6840; 95% CI: −5.5392 to −0.6476; *p*‐value: 0.010398)]and gradual increase in trends in both races between 2010 and 2018 and reaching to their maximum height in the year 2021 [(APC value for Black: 9.3257; 95% CI: 6.2949 to 11.4277; *p*‐value: 0.000800); (APC value for Hispanics: 13.0861; 95% CI: 9.2822 to 15.6965; *p*‐value: < 0.000001)] followed by marked fall in mortality rates from 2021 to 2023 in Black as well as Hispanics [(APC value for Black: −4.4613; 95% CI: −7.8607 to −0.5990; *p*‐value: 0.024795), (APC value for Hispanics: −8.9850; 95% CI: −13.0218 to −4.9048; *p*‐value: 0.0008000)]. The other two races, American Indian or Alaska Native and Asian or Other Pacific Islander, show more distinguished mortality rate trends between 1999 and 2023. American Indian or Alaska Native shows a consistent increase in death rates from 1999 to 2020 (APC: 1.6841; 95% CI: 1.2821 to 2.2504; *p*‐value: < 0.000001) then a rapid fall from 2020 to 2023 (APC: −7.7567; 95% CI: −13.8100 to −3.2961; *p*‐value: 0.001600) while in Asian or Other Pacific Islander there was a gradual reduction in mortality from 1999 to 2018 (APC: −0.3402: 95% CI: −0.9543 to 0.2499; *p*‐value: 0.188762) following which there was rapid upstroke in graph showing increase in mortality rate between 2018 and 2021 (APC: 9.8413; 95% CI: 0.0820 to 12.0977; *p*‐value: 0.049590) then again a more steeper but insignificant fall in trend of deaths rate from 2021 to 2023 (APC: −5.6383; 95% CI: −11.4694 to 2.6041; *p*‐value: 0.090382). These findings are presented in Figure 3, Tables S3 and S5 and in Figure S3.

### 3.4 Trends for Arrhythmia‐Related AAMR Stratified by Geographic Region

A significant number of disparities were observed in AAMR when stratified by states, starting from Hawaii with an AAMR value of 88.2 to Oregon with an AAMR of 182.45. States that fell in the category of the top 90th percentile were (Oregon, South Carolina, Oklahoma, Vermont, and Nebraska), having double the AAMR as compared to those that fell into the lower 10th percentile namely, (Nevada, Florida, New York, New Mexico, and Hawaii) (Figure S4, Table S6).

We also analyzed disparities in trends of mortality rate based on census region, in which we found highest value for overall AAMR between 1999 and 2023 in Mid‐west region (overall AAMR: 122;988; 95% CI: 121.88 to 124.068) followed by West region (AAMR: 112.608; 95% CI: 111.508 to 113.68) while South region and Northeast region comes next [(AAMR for South: 111.148; 95% CI: 110.312 to 111.988), (AAMR for Northeast: 106.196; 95% CI: 105.112 to 107.28)]. Starting with 1999 on analysis we observed decline in all four regions however, in South and West regions there was a decrease in mortality rate till 2009 [(APC for South: −1.0901; 95% CI: −1.9117 to −0.5242; *p*‐value: < 0.000001), (West's APC: −0.4686; 95% CI: −2.2294 to 0.1448; *p*‐value: 0.125975)] while Mid‐west and Northeast shows decline till 2010 and 2007 respectively with the APC Value of [(APC for Mid ‐west:: −0.8740; 95% CI: −1.6635 to −0.4258; *p*‐value: < 0.000001), (APC for Northeast region: −1.9367; 95% CI: −3.5035 to −1.1841; *p*‐value: < 0.000001)], then all of them observed a significant rise in mortality trends i.e. from 2009 to 2018 in the South and west regions [(APC for South: 1.8088; 95% CI: 1.0429 to 2.6204; p‐value: < 0.000001), (APC for West: 1.7426; 95% CI: 0.8233 to 2.7424; *p*‐value: 0.001200)] reaching their peaks in 2021 with APC values of [(APC for South: 10.2179; 95% CI: 8.0380 to 11.7082; *p*‐value: < 0.000001), (APC for West: 7.8877; 95% CI: 5.5376 to 9.3563; *p*‐value: < 0.000001)] while from 2010 to 2018 in Mid‐west and in between 2007 and 2018 in Northeast [(APC for Mid ‐west: 1.9401; 95% CI: 0.9398 to 2.8928; *p*‐value: < 0.000001),(APC for Northeast: 0.9612; 95% CI: 0.3204 to 1.5877; *p*‐value: 0.011198)] reaching their peaks in the year 2021 [(APC for Mid‐ west: 8.6060; 95% CI: 6.3459 to 9.9878; *p*‐value: < 0.000001), APC for Northeast: 7.5452; 95% CI: 5.0748 to 8.9982; *p*‐value: 0.001200) followed by recent decline in fatality rate in 2021 to 2023 in all four regions with an APC values of [(APC for Mid‐west: −5.8504; 95% CI: −8.7751 to −3.1829; *p*‐value: < 0.000001), (APC for Northeast: −4.3922; 95% CI: −7.3532 to −1.0508; *p*‐value: 0.013197), (APC for South: −3.5872; 95% CI: −6.0396 to −1.1652; *p*‐value: 0.006799), (APC for West: −3.3536; 95% CI: −6.1293 to 0.0057; *p*‐value: 0.050390)]. (Figures S4 and S5; Tables S3 and S7).

While analyzing AAMR trends in 1999 to 2020 in accordance to urbanization we observed non‐metropolitans area with consistently higher AAMR values than metropolitan areas (Overall AAMR value for Non metropolitan areas: 123.8; Overall AAMR for metropolitan areas: 106.8). Beginning with 1999 both of them observed a decline in death rates till 2009 however, descend in fatality rate was more in metropolitan areas in comparison to non‐metropolitans [(APC value for Metropolitan: −1.1363; 95% CI: −0.9833 to −0.6463; *p*‐value: 0.005599), (APC value for Nonmetropolitan: −0.5394; 95% CI: −1.1689 to −0.0682; *p*‐value: 0.030794)] followed by substantial growth in death rates from 2009 to 2018 [(APC value for Nonmetropolitan: 2.4708; 95% CI: 1.7124 to 3.1046; *p*‐value: 0.000400), (APC value for metropolitan: 1.5704; 95% CI: 0.4321 to 2.3180; *p*‐value: 0.026395)] following which there is a maximum spike in mortality rates between 2018 and 2020, the spike is more pronounced in nonmetropolitan areas than metropolitan areas[(APC value for nonmetropolitan areas: 9.0422; 95% CI: 5.4684 to 10.9743; *p*‐value: < 0.000001), (APC value for metropolitan areas: 7.4825; 95% CI: 3.5702 to 9.5339; *p*‐value: < 0.000001)]. (Figures S6 and S7; Tables S3 and S8).

### 3.5 Subgroup by Types of Arrhythmia

Subgroup analyses also revealed significant disparities in the types of different arrhythmias. However, the highest number of mortalities, 1,26,048, is observed when cardiac arrhythmia is unspecified, referring to cases in which the death certificate did not provide further specification of the arrhythmia subtype. This huge number of mortalities accounts for **59%** of arrhythmia‐related mortality in all ages. Among them are unspecified tachycardia‐related deaths, which account for 110,399 deaths and making **5.7%** of all arrhythmia‐related deaths. In mortalities where the type of arrhythmia is specified, 276,738 mortalities from ventricular fibrillation and flutter were observed to be the most, making **14.5%** of deaths among all arrhythmia‐related deaths. It is followed by mortality from ventricular tachycardia, which accounts for 150,859 deaths, making up **7.9%** of total arrhythmias‐related mortalities (Table 2).

### 3.6 Comparison of Arrhythmia and CVD‐Related Mortality

Using the same age‐standardization method (based on the 2000 U.S. standard population), overall cardiovascular mortality (ICD‐10 codes I00–I99) declined from an AAMR of 681.8 per 100,000 in 1999 to 423.2 per 100,000 in 2023. In contrast, arrhythmia‐related mortality increased from 111.4 per 100,000 in 1999 to 137.3 per 100,000 in 2023. This shift means that arrhythmia‐related deaths accounted for roughly 16% of all cardiovascular deaths in 1999 (111.4/681.8) but rose to nearly 32% in 2023 (137.3/423.2). These findings indicate that while overall CVD mortality has decreased substantially over the past two decades, the relative contribution of arrhythmias to cardiovascular deaths has more than doubled, highlighting an emerging public health concern (Figure S8).

## 4 Discussion

This 25‐year analysis of arrhythmia‐related mortality in adults aged 35 years and older reveals critical trends and disparities in the United States. The age‐adjusted mortality rate (AAMR) declined from 1999 to 2009, followed by a significant increase until 2018, peaking in 2021, and then sharply decreasing through 2023. These trends suggest that while advancements in cardiovascular care may have driven earlier improvements, subsequent periods of rising mortality could reflect increased comorbidity burdens and shifting healthcare practices. By the end of the study period, nonmetropolitan areas, NH White populations, and males consistently demonstrated higher mortality rates compared to their counterparts, underscoring persistent disparities in arrhythmia‐related outcomes. Moreover, the findings from our subgroup analyses underscore significant disparities in the burden of arrhythmia‐related mortality, emphasizing the critical role of classification and diagnostic specificity. However, significant disparities persisted across gender, race, place of death, and geographic lines. Females accounted for **51%** of DCM‐related deaths, with higher AAMR compared to males. (Table 1).

**Table 1:** Number of overall deaths and AAMR in different sets of populations.

Variable	Arrhythmia deaths (*n*)	AAMRs (95% CI) per 100,000
Overall population	5,050,271 (100%)	113.3 (112.8–113.8)
**Sex**		
Male	2,479,122 (49.1%)	137.2 (136.3–138.1)
Female	2,571,149 (50.9%)	95.3 (94.7–95.9)
**Census region**		
Northeast	25,076 (14.8%)	106.2 (105.1–107.3)
Midwest	1,237,272 (24.4%)	123.0 (121.9–124.1)
South	1,793,413 (35.5%)	111.1 (110.3–112.0)
West	1,077,114 (21.3%)	112.6 (111.5–113.7)
**Race/Ethnicity**		
American Indian or Alaska Native	22,218 (0.4%)	87.5 (81.0–94.0)
Asian or Pacific Islander	96,585 (1.9%)	57.4 (55.4–59.4)
Black or African American	420,147 (8.3%)	105.5 (103.9–107.2)
White	4,332,217 (85.7%)	119.4 (118.8–119.9)
Hispanic or Latino	220,144 (4.3%)	67.6 (66.1–69.2)
**Urbanization (1999–2020)**		
Metropolitan	140,233 (83.1%)	106.8 (106.7–106.9)
Nonmetropolitan	3,313,312 (80.09%)	123.8 (123.5–124.1)
**Place of death**		
Medical facility	2,377,858 (49%)	—
Decedent's home	1,188,929 (24.56%)	—
Nursing home/health care facilities	1,118,563 (23.05%)	—
Hospice facility	181,978 (3.75%)	—

**Table 2:** Number of overall deaths in different types of arrhythmia.

Subtypes		
Condition	Deaths	Percentage (%)
Cardiac arrhythmia, unspecified	1,126,048	59.7
Ventricular fibrillation and flutter	276,738	14.5
Ventricular tachycardia	150,859	7.9
Tachycardia, unspecified	110,399	5.7
Other Types	242,197	12.8

Multiple factors influence the overall trends in mortality. The decline in AAMR from 1999 to 2009 parallels advances in cardiovascular treatments, including the widespread use of implantable cardioverter‐defibrillators and antiarrhythmic drugs [15]. However, the subsequent rise in AAMR from 2009 onward could be attributed to the increasing prevalence of arrhythmia risk factors such as hypertension, obesity, and diabetes in the aging U.S. population [16]. Notably, the 2021 peak coincided with the COVID‐19 pandemic, which disrupted access to routine care and exacerbated cardiovascular outcomes [17]. Sex‐specific disparities persisted throughout the study, with males demonstrating higher AAMRs than females. This disparity may be attributed to a greater prevalence of coronary artery disease and ventricular arrhythmias in men [18], compounded by the potential underdiagnosis of arrhythmias in women due to atypical symptom presentation [19].

Racial disparities were a significant finding, with NH Whites exhibiting the highest mortality rates followed by NH Black populations, while Asian/Pacific Islanders had the lowest AAMRs. These differences align with previous evidence that points to structural inequities in healthcare access, socioeconomic challenges, and racial bias in healthcare delivery [20]. NH Black populations, for example, face barriers to early diagnosis and treatment of arrhythmias, and higher rates of comorbid conditions such as hypertension further exacerbate outcomes [21]. Meanwhile, Hispanic populations exhibited a pronounced mortality reduction between 2021 and 2023, which may reflect recent improvements in access to care among this demographic. Disparities in arrhythmia prevalence and outcomes among racial groups could also be partially attributed to differences in genetic predisposition and social determinants of health [22]. Addressing these inequities will require a multifaceted approach integrating improved access to culturally competent care, enhanced screening in high‐risk populations, and public health strategies targeting underlying comorbidities.

Geographic disparities revealed higher mortality rates in nonmetropolitan areas and the Midwest than in metropolitan regions and other census divisions. Limited access to specialized cardiology care, fewer healthcare facilities, and socioeconomic challenges in rural areas contribute to these discrepancies [23]. States with the highest mortality rates, such as Oregon and South Carolina, consistently reported double the AAMR of states like Hawaii and Nevada, emphasizing the impact of regional healthcare infrastructure and preventive care availability. The pronounced rural‐urban divide further underscores the need for targeted interventions in underserved areas.

Addressing these disparities requires comprehensive public health strategies. Expanding access to arrhythmia management services in nonmetropolitan areas, promoting early detection through population‐based screening, and integrating culturally tailored care for high‐risk racial and ethnic groups could mitigate disparities [24]. Additionally, national policies should emphasize improving healthcare infrastructure in rural regions and increasing public awareness about arrhythmia prevention and management [25]. Leveraging telemedicine platforms to bridge gaps in specialist access, particularly in the aftermath of the COVID‐19 pandemic, could significantly enhance care delivery [26].

Cultural competence training and tailored interventions are essential to reduce barriers faced by NH Black, Hispanic, and other minority populations in accessing arrhythmia care. Additionally, addressing gender‐specific needs through increased awareness of atypical arrhythmia presentations in women and improving cardiovascular care resources for men may further reduce disparities [27].

Notably, the largest proportion of deaths (59%) was attributed to unspecified cardiac arrhythmias, highlighting a substantial gap in precise arrhythmia diagnosis and reporting. This diagnostic ambiguity likely impedes targeted clinical interventions and resource allocation, underscoring the need for enhanced diagnostic tools and standardized reporting protocols in clinical practice [28, 29]. Among specified arrhythmias, ventricular fibrillation and flutter emerged as the leading contributors to arrhythmia‐related mortality (14.5%), followed by ventricular tachycardia (7.9%) [30, 31]. These findings align with the known lethality of ventricular arrhythmias, particularly in the absence of timely medical intervention.

These disparities point to critical areas for intervention, including improving diagnostic precision, promoting early detection, and enhancing treatment strategies for high‐risk arrhythmias, particularly ventricular fibrillation and tachycardia. Future research should focus on understanding the underlying factors contributing to these disparities, such as access to care, advancements in diagnostic technologies, and the role of comorbid conditions in influencing arrhythmia‐related mortality [32].

### 4.1 Limitation

This study has several limitations. First, the reliance on death certificates introduces the potential for misclassification of arrhythmia‐related deaths. The CDC WONDER database lacks clinical details such as arrhythmia subtypes, severity, and treatment history, precluding granular analysis. Second, the study's age cutoff of 35 years may underrepresent younger populations at risk of arrhythmias due to congenital or inherited conditions. Third, the study's observational nature precludes causal inferences, and the absence of socioeconomic data limits the exploration of underlying factors driving disparities. Additionally, the study's observational nature precludes causal inferences, and the impact of evolving diagnostic practices and reporting standards remains unaccounted. Finally, the impacts of the COVID‐19 pandemic on arrhythmia mortality are incompletely captured, necessitating further research to evaluate post‐pandemic trends.

## 5 Conclusion

This study highlights significant temporal, demographic, and geographic disparities in arrhythmia‐related mortality from 1999 to 2023. The findings emphasize the critical need for targeted public health initiatives to address disparities based on sex, race, and geography while improving access to arrhythmia care in underserved populations. Future research should incorporate clinical and socioeconomic data to refine interventions and reduce arrhythmia mortality across diverse populations.

## Author Contributions

**Bazil Azeem:** conceptualization, project administration, validation, visualization, writing – original draft, writing – review and editing. **Laiba Khurram:** conceptualization, writing – original draft, writing – review and editing. **Bakhtawar Sharaf:** writing – original draft, writing – review and editing. **Arwa Khan:** data curation, writing – original draft, writing – review and editing. **Ayesha Habiba:** formal analysis, writing – original draft, writing – review and editing. **Rabia Asim:** formal analysis, writing – original draft, writing – review and editing. **Muskan Khelani:** methodology, writing – original draft, writing – review and editing. **Hamza Ali:** visualization, writing – original draft, writing – review and editing. **Abdul Hadi Ansari:** writing – original draft, writing – review and editing. **Tazheen Saleh Muhammad:** writing – original draft, writing – review and editing. **Muhammad Abdullah Naveed:** writing – original draft, writing – review and editing. **Aalaa Saleh:** project administration, writing – original draft, writing – review and editing. **Hamza Ashraf:** writing – original draft, writing – review and editing.

## Conflicts of Interest

The authors declare no conflicts of interest.

## Supporting information

Supporting information.

## Data Availability

The data that support the findings of this study are openly available in CDC WONDER at https://wonder.cdc.gov/, reference number N/A. This paper has not been previously published and is not currently under consideration by another journal.
