# Supplementary material for: Unmasking Arrhythmia Mortality: A 25‐Year Analysis of Trends and Disparities in the United States (1999–2023)
Source: Clin Cardiol. 2025 Mar 4;48(3):e70109. doi: 10.1002/clc.70109 (PMC11877330; doi:10.1002/clc.70109)
Supplement: Supplementary file 1 — Supporting information. [file CLC-48-e70109-s001.docx]

**Supplementary Materials**

| **Supplemental Table 1: Arrhythmia–related Deaths, Stratified by Sex and Race, in Adults of age ≥35 in the United States, 1999 to 2023** | | | | | | | | | | | | | |
| --- | --- | --- | --- | --- | --- | --- | --- | --- | --- | --- | --- | --- | --- |
| **Deaths** | | | | | | | | | | | | | |
| **Year** | **Overall** | | **Women** | **Men** | | **NH White** | **NH Black or African American** | **NH Asian or Pacific Islander** | **NH American Indian or Alaska Native** | | **Hispanic or Latino** | | **Population** |
| **1999** | 154918 | | 80895 | 74023 | | 134650 | 13417 | 1722 | 469 | | 4303 | | 140230363 |
| **2000** | 148403 | | 78866 | 69537 | | 129566 | 12270 | 1609 | 447 | | 4142 | | 142092916 |
| **2001** | 157449 | | 83748 | 73701 | | 136217 | 13446 | 1977 | 480 | | 4982 | | 144833606 |
| **2002** | 160120 | | 85246 | 74874 | | 138796 | 13370 | 2193 | 456 | | 4918 | | 146858382 |
| **2003** | 159091 | | 84830 | 74261 | | 137558 | 13424 | 2126 | 511 | | 5159 | | 148846634 |
| **2004** | 154231 | | 82007 | 72224 | | 132938 | 13208 | 2164 | 519 | | 5184 | | 150938828 |
| **2005** | 162513 | | 86751 | 75762 | | 139981 | 13637 | 2406 | 525 | | 5727 | | 153292737 |
| **2006** | 160541 | | 85842 | 74699 | | 138342 | 13291 | 2542 | 583 | | 5582 | | 155624180 |
| **2007** | 161434 | | 85909 | 75525 | | 139369 | 13304 | 2554 | 584 | | 5472 | | 157690314 |
| **2008** | 166412 | | 88753 | 77659 | | 143561 | 13378 | 2743 | 562 | | 5904 | | 159587617 |
| **2009** | 164497 | | 86839 | 77658 | | 141582 | 13227 | 2833 | 618 | | 5990 | | 161383674 |
| **2010** | 171628 | | 90141 | 81487 | | 147582 | 13613 | 3007 | 682 | | 6495 | | 162828035 |
| **2011** | 180699 | | 95147 | 85552 | | 155309 | 14225 | 3243 | 716 | | 6973 | | 164802438 |
| **2012** | 187112 | | 97628 | 89484 | | 159979 | 15070 | 3370 | 768 | | 7509 | | 166516716 |
| **2013** | 196581 | | 101644 | 94937 | | 167686 | 15826 | 3842 | 822 | | 8024 | | 168240727 |
| **2014** | 201554 | | 103236 | 98318 | | 171225 | 16271 | 3921 | 879 | | 8753 | | 170292776 |
| **2015** | 214209 | | 109329 | 104880 | | 181594 | 17084 | 4209 | 1010 | | 9607 | | 172416615 |
| **2016** | 219645 | | 110726 | 108919 | | 185223 | 17812 | 4694 | 1034 | | 10351 | | 173964174 |
| **2017** | 232375 | | 116076 | 116299 | | 195698 | 18874 | 5037 | 1099 | | 11139 | | 176104659 |
| **2018** | 241146 | | 119483 | 121663 | | 202553 | 19685 | 5375 | 1176 | | 11861 | | 177613416 |
| **2019** | 249666 | | 122498 | 127168 | | 209425 | 20425 | 5690 | 1161 | | 12548 | | 179040846 |
| **2020** | 292564 | | 140558 | 152006 | | 240028 | 25924 | 7094 | 1543 | | 17472 | | 180565367 |
| **2021** | 309290 | | 146294 | 162996 | | 271984 | 27014 | 7435 | 1590 | | 18578 | | 182743307 |
| **2022** | 308,741 | | 147,727 | 161,014 | | 271,684 | 26,634 | 7,572 | 1,537 | | 17,295 | | 184007299 |
| **2023** | 295452 | | 140976 | 154476 | | 259687 | 25718 | 7227 | 1447 | | 16176 | | 184007299 |
| **Total** | **5,050,271** | | **2,571,149** | **2,479,122** | | **4,332,217** | **420,147** | **96,585** | **21,218** | | **220,144** | | 4,104,522,925 |
| NH: Non-Hispanics | | | | | | | | | | | | | |
| **Supplemental Table 2: Arrhythmia–related Mortality, Stratified by Place of Death in Adults of age ≥35 in the United States, 1999 to 2023** | | | | | | | | | | | | | |
| **Deaths** | | | | | | | | | | | | | |
| **Year** | | **Medical Facility** | | | **Nursing Home/Long-term Care Facility** | | | | | **Hospices** | | **Home** | |
| **1999** | | 88,554 | | | 35986 | | | | | - | | 26890 | |
| **2000** | | 82,893 | | | 35385 | | | | | - | | 26271 | |
| **2001** | | 86,673 | | | 38386 | | | | | - | | 27980 | |
| **2002** | | 86,528 | | | 39336 | | | | | - | | 29324 | |
| **2003** | | 84,170 | | | 39032 | | | | | 174 | | 30029 | |
| **2004** | | 80,283 | | | 37768 | | | | | 354 | | 30032 | |
| **2005** | | 83,449 | | | 40321 | | | | | 1423 | | 31628 | |
| **2006** | | 81,386 | | | 39530 | | | | | 2206 | | 31823 | |
| **2007** | | 80,802 | | | 39386 | | | | | 3066 | | 32484 | |
| **2008** | | 81,970 | | | 39720 | | | | | 3734 | | 33288 | |
| **2009** | | 78,740 | | | 38580 | | | | | 3811 | | 34029 | |
| **2010** | | 82,060 | | | 40470 | | | | | 5030 | | 37350 | |
| **2011** | | 84,784 | | | 42753 | | | | | 6100 | | 39693 | |
| **2012** | | 84,808 | | | 44004 | | | | | 7599 | | 42786 | |
| **2013** | | 88,108 | | | 45903 | | | | | 7757 | | 46079 | |
| **2014** | | 89,525 | | | 46764 | | | | | 9035 | | 48420 | |
| **2015** | | 93,692 | | | 49546 | | | | | 10834 | | 52230 | |
| **2016** | | 95,716 | | | 49272 | | | | | 11696 | | 54568 | |
| **2017** | | 99,486 | | | 51937 | | | | | 12980 | | 58887 | |
| **2018** | | 102,729 | | | 53256 | | | | | 13586 | | 62158 | |
| **2019** | | 104,835 | | | 53438 | | | | | 15396 | | 65977 | |
| **2020** | | 122,938 | | | 56191 | | | | | 15500 | | 85328 | |
| **2021** | | 138,254 | | | 51,399 | | | | | 16,666 | | 89,956 | |
| **2022** | | 133,916 | | | 54,122 | | | | | 17,193 | | 89,469 | |
| **2023** | | 123,805 | | | 55,895 | | | | | 18,098 | | 85,371 | |
| **Total** | | **2,360,104** | | | **1,118,380** | | | | | **182,238** | | **1,192,050** | |

| **Supplemental Table 3: Annual percent change (APC) of Arrhythmia-related Age-Adjusted Mortality Rates per 100,000 in Adults aged ≥35 in the United States, 1999 to 2023** | | |
| --- | --- | --- |
| **Year Interval** | **APC (95% CI)** | **P value** |
| **Overall** | | |
| 1999 to 2009 | -1.0* (-2.4 to -0.4) | 0.003199 |
| 2009 to 2018 | 1.7* (0.8 to 2.6) | 0.003199 |
| 2018 to 2021 | 8.6* (6.1 to 10.0) | 0.0004 |
| 2021 to 2023 | -3.9* (-7.6 to -0.2) | 0.043991 |
| **Men** | | |
| 1999 to 2009 | -1.4* (-2.4 to -0.9) | < 0.000001 |
| 2009 to 2018 | 2.0* (1.1 to 2.7) | < 0.000001 |
| 2018 to 2021 | 9.6* (7.3 to 11.0) | < 0.000001 |
| 2021 to 2023 | -3.7* (-6.4 to -1.1) | 0.016397 |
| **Women** | | |
| 1999 to 2009 | -0.7* (-1.9 to -0.2) | 0.004399 |
| 2009 to 2018 | 1.3* (0.5 to 2.2) | 0.0012 |
| 2018 to 2021 | 8.1* (6.0 to 9.5) | < 0.000001 |
| 2021 to 2023 | -4.6* (-7.2 to -2.1) | < 0.000001 |
| **NH White** | | |
| 1999 to 2010 | -0.8* (-1.4 to -0.3) | 0.0004 |
| 2010 to 2018 | 2.1* (1.4 to 2.7) | < 0.000001 |
| 2018 to 2021 | 7.9* (6.1 to 9.0) | < 0.000001 |
| 2021 to 2023 | -4.8* (-6.9 to -2.8) | < 0.000001 |
| **NH Black or African American** | | |
| 1999 to 2009 | -1.9* (-3.3 to -1.2) | 0.004399 |
| 2009 to 2018 | 1.0 (-0.4 to 2.6) | 0.145171 |
| 2018 to 2021 | 9.3* (6.3 to 11.4) | 0.0008 |
| 2021 to 2023 | -4.5* (-7.9 to -0.6) | 0.024795 |
| **NH American Indian or Alaska Native** | | |
| 1999 to 2020 | 1.7* (1.3 to 2.3) | < 0.000001 |
| 2020 to 2023 | -7.8* (-13.8 to -3.3) | 0.0016 |
| **Hispanic or Latino** | | |
| 1999 to 2010 | -1.7* (-5.5 to -0.6) | 0.010398 |
| 2010 to 2018 | 1.7 (-0.1 to 4.3) | 0.068786 |
| 2018 to 2021 | 13.1* (9.3 to 15.7) | < 0.000001 |
| 2021 to 2023 | -9.0* (-13.0 to -4.9) | 0.0008 |
| **NH Asian or Pacific Islander** | | |
| 1999 to 2018 | -0.3 (-1.0 to 0.2) | 0.188762 |
| 2018 to 2021 | 9.8* (0.1 to 12.1) | 0.04959 |
| 2021 to 2023 | -5.6 (-11.5 to 2.6) | 0.090382 |
| **Nonmetropolitan areas** | | |
| 1999 to 2009 | -0.5* (-1.2 to -0.1) | 0.030794 |
| 2009 to 2018 | 2.5* (1.7 to 3.1) | 0.0004 |
| 2018 to 2020 | 9.0* (5.5 to 11.0) | < 0.000001 |
| **Metropolitan area** | | |
| 1999 to 2009 | -1.1* (-2.0 to -0.6) | 0.005599 |
| 2009 to 2018 | 1.6* (0.4 to 2.3) | 0.026395 |
| 2018 to 2020 | 7.5* (3.6 to 9.5) | < 0.000001 |
| **Northeast region** | | |
| 1999 to 2007 | -1.9* (-3.5 to -1.2) | < 0.000001 |
| 2007 to 2018 | 1.0* (0.3 to 1.6) | 0.011198 |
| 2018 to 2021 | 7.5* (5.1 to 9.0) | 0.0012 |
| 2021 to 2023 | -4.4* (-7.4 to -1.1) | 0.013197 |
| **Midwest region** | | |
| 1999 to 2010 | -0.9* (-1.7 to -0.4) | < 0.000001 |
| 2010 to 2018 | 1.9* (0.9 to 2.9) | < 0.000001 |
| 2018 to 2021 | 8.6* (6.3 to 10.0) | < 0.000001 |
| 2021 to 2023 | -5.9* (-8.8 to -3.2) | < 0.000001 |
| **South region** | | |
| 1999 to 2009 | -1.1* (-1.9 to -0.5) | < 0.000001 |
| 2009 to 2018 | 1.8* (1.0 to 2.6) | < 0.000001 |
| 2018 to 2021 | 10.2* (8.0 to 11.7) | < 0.000001 |
| 2021 to 2023 | -3.6* (-6.0 to -1.2) | 0.006799 |
| **West region** | | |
| 1999 to 2009 | -0.5 (-2.2 to 0.1) | 0.125975 |
| 2009 to 2018 | 1.7* (0.8 to 2.7) | 0.0012 |
| 2018 to 2021 | 7.9* (5.5 to 9.4) | < 0.000001 |
| 2021 to 2023 | -3.4 (-6.1 to 0.0) | 0.05039 |
| APC = annual percent change; NH = non-Hispanic; * Indicates that the annual percentage change (APC) is significantly different from zero at α = 0.05. | | |

| **Supplemental Table 4: Overall and Sex‐Stratified Arrhythmia–related Age-Adjusted Mortality Rates per 1,00,000 in Adults aged ≥35 in the United States, 1999 to 2020** | | | |
| --- | --- | --- | --- |
| **Age-Adjusted Rate (95% CI)** | | | |
| **Year** | **Men** | **Women** | **Overall** |
| **1999** | 138.8 (137.8–139.8) | 92.1 (91.5–92.8) | 111.4 (110.9–112) |
| **2000** | 129.2 (128.2–130.1) | 88.5 (87.8–89.1) | 105.4 (104.8–105.9) |
| **2001** | 134 (133–135) | 92.8 (92.1–93.4) | 109.9 (109.4–110.5) |
| **2002** | 133.8 (132.8–134.8) | 93.3 (92.7–94) | 110.2 (109.7–110.7) |
| **2003** | 130 (129.1–131) | 91.6 (90.9–92.2) | 107.7 (107.1–108.2) |
| **2004** | 124 (123–124.9) | 87.6 (87–88.2) | 102.9 (102.4–103.4) |
| **2005** | 127.1 (126.2–128) | 90.9 (90.3–91.5) | 106.2 (105.6–106.7) |
| **2006** | 121.9 (121–122.8) | 88.4 (87.8–89) | 102.7 (102.2–103.2) |
| **2007** | 120.5 (119.6–121.3) | 86.8 (86.2–87.4) | 101.1 (100.6–101.6) |
| **2008** | 120.8 (119.9–121.6) | 88 (87.4–88.6) | 102 (101.5–102.5) |
| **2009** | 117.7 (116.9–118.6) | 84.7 (84.1–85.3) | 98.8 (98.4–99.3) |
| **2010** | 121.2 (120.4–122.1) | 86.5 (85.9–87.1) | 101.3 (100.9–101.8) |
| **2011** | 122.8 (121.9–123.6) | 89 (88.4–89.6) | 103.6 (103.2–104.1) |
| **2012** | 124.8 (123.9–125.6) | 89.5 (89–90.1) | 104.7 (104.2–105.2) |
| **2013** | 128.2 (127.4–129.1) | 91.5 (90.9–92.1) | 107.4 (106.9–107.9) |
| **2014** | 129.1 (128.3–129.9) | 91.3 (90.7–91.9) | 107.7 (107.2–108.2) |
| **2015** | 133.8 (132.9–134.6) | 94.9 (94.3–95.5) | 111.9 (111.4–112.4) |
| **2016** | 135.4 (134.6–136.2) | 94.5 (94–95.1) | 112.4 (111.9–112.9) |
| **2017** | 140.7 (139.9–141.5) | 97.3 (96.7–97.8) | 116.2 (115.8–116.7) |
| **2018** | 143.1 (142.3–143.9) | 98.3 (97.7–98.8) | 117.9 (117.4–118.4) |
| **2019** | 145.7 (144.8–146.5) | 99.3 (98.8–99.9) | 119.7 (119.2–120.1) |
| **2020** | 170.2 (169.4–171.1) | 112.5 (111.9–113.1) | 137.8 (137.3–138.3) |
| **2021** | 187.4 (186.5–188.3) | 124.2 (123.5–124.8) | 152 (151.5–152.6) |
| **2022** | 178.4 (177.5–179.2) | 116.8 (116.2–117.4) | 143.4 (142.9–143.9) |
| **2023** | 171.6 (170.7–172.5) | 111.4 (110.9–112) | 137.3 (136.8–137.8) |
| **Total** | **137.2 (136.3–138.1)** | **95.3 (94.7–95.9)** | **113.3 (112.8–113.8)** |

| **Supplemental Table 5: Arrhythmia–related Age-Adjusted Mortality Rates per 1,00,000 in Adults aged ≥35 in the United States, 1999 to 2023** | | | | | |
| --- | --- | --- | --- | --- | --- |
| **Age-Adjusted Rate (95% CI)** | | | | | |
| **Year** | **NH White** | **NH Black or African American** | **NH American Indian or Alaska Native** | **Hispanic or Latino** | **NH Asian or Pacific Islander** |
| **1999** | 114 (113.4–114.6) | 115.5 (113.5–117.4) | 84.9 (76.7–93.1) | 68.3 (66.2–70.4) | 59.5 (56.5–62.4) |
| **2000** | 108.5 (107.9–109.1) | 104.5 (102.6–106.4) | 75.4 (68.1–82.8) | 62.9 (60.9–64.9) | 52.8 (50.1–55.5) |
| **2001** | 112.6 (112–113.2) | 112.6 (110.6–114.5) | 78.2 (70.8–85.6) | 71.8 (69.7–73.9) | 60.3 (57.5–63) |
| **2002** | 113.4 (112.8–114) | 110.4 (108.5–112.3) | 75.3 (68–82.6) | 67.8 (65.8–69.8) | 61.8 (59.1–64.5) |
| **2003** | 110.9 (110.3–111.5) | 108.9 (107–110.8) | 82 (74.4–89.5) | 67.2 (65.3–69.1) | 57.5 (55–60.1) |
| **2004** | 106 (105.5–106.6) | 105 (103.2–106.8) | 79.9 (72.6–87.3) | 64.4 (62.6–66.2) | 55.1 (52.7–57.5) |
| **2005** | 109.8 (109.2–110.4) | 105.8 (104–107.6) | 75.9 (69–82.9) | 67.1 (65.2–68.9) | 56.7 (54.4–59.1) |
| **2006** | 106.5 (106–107.1) | 100.2 (98.5–102) | 83.5 (76.2–90.7) | 62.5 (60.8–64.2) | 56.9 (54.6–59.2) |
| **2007** | 105.5 (105–106.1) | 98.1 (96.4–99.8) | 82.3 (75.2–89.3) | 58.3 (56.7–59.9) | 53.3 (51.2–55.4) |
| **2008** | 106.8 (106.2–107.4) | 96.2 (94.5–97.8) | 76.5 (69.8–83.3) | 59.4 (57.8–61) | 55.2 (53.1–57.3) |
| **2009** | 103.8 (103.3–104.4) | 92.3 (90.7–93.9) | 82.1 (75.3–89) | 57.1 (55.6–58.6) | 53.5 (51.5–55.5) |
| **2010** | 106.7 (106.2–107.3) | 93.3 (91.6–94.9) | 89.3 (82.2–96.4) | 59.4 (57.9–60.9) | 54.6 (52.6–56.6) |
| **2011** | 109.8 (109.3–110.4) | 94.1 (92.6–95.7) | 88.4 (81.6–95.2) | 59 (57.6–60.4) | 54 (52.1–55.9) |
| **2012** | 111.1 (110.5–111.6) | 96 (94.4–97.6) | 87.8 (81.3–94.4) | 59.8 (58.4–61.2) | 52.3 (50.5–54.1) |
| **2013** | 114.5 (113.9–115) | 97.2 (95.6–98.7) | 87.3 (81–93.6) | 60.2 (58.9–61.6) | 55.2 (53.4–57) |
| **2014** | 115.2 (114.6–115.8) | 96.8 (95.3–98.4) | 90 (83.8–96.3) | 62 (60.6–63.3) | 52.4 (50.7–54.1) |
| **2015** | 120.4 (119.8–120.9) | 98.3 (96.8–99.9) | 97.6 (91.3–103.8) | 63.8 (62.5–65.1) | 52.5 (50.9–54.1) |
| **2016** | 120.9 (120.4–121.5) | 99.5 (98–101) | 96.7 (90.6–102.8) | 65.4 (64.1–66.7) | 55.5 (53.9–57.1) |
| **2017** | 125.9 (125.3–126.4) | 101.6 (100.1–103.1) | 99.5 (93.4–105.6) | 66.6 (65.3–67.9) | 55.6 (54.1–57.2) |
| **2018** | 127.9 (127.4–128.5) | 102.9 (101.4–104.3) | 98.7 (92.9–104.5) | 67.4 (66.2–68.7) | 56.4 (54.9–57.9) |
| **2019** | 130.4 (129.8–130.9) | 103.5 (102–104.9) | 93.6 (88.1–99.2) | 68.6 (67.3–69.8) | 56.6 (55.1–58.1) |
| **2020** | 148.1 (147.5–148.7) | 127.8 (126.2–129.4) | 118.7 (112.7–124.8) | 90.6 (89.2–92) | 66.8 (65.3–68.4) |
| **2021** | 160.1 (159.4–160.7) | 130.8 (129.2–132.4) | 95.3 (90.4–100.2) | 95.6 (94.1–97) | 70.8 (69.1–72.4) |
| **2022** | 150.9 (150.3–151.5) | 125.3 (123.7–126.8) | 87.2 (82.8–91.7) | 85.5 (84.2–86.9) | 66.5 (65–68) |
| **2023** | 144.3 (143.7–144.8) | 121.4 (119.9–122.9) | 82.2 (77.9–86.6) | 80.5 (79.2–81.7) | 63.7 (62.3–65.2) |
| **Total** | **119.4 (118.8–119.9)** | **105.5 (103.9–107.2)** | **87.5 (81.0–94.0)** | **67.6 (66.1–69.2)** | **57.4 (55.4–59.4)** |
| NH = Non-Hispanic. | | | | | |

| **Supplemental Table 6: Arrhythmia–related Age-Adjusted Mortality Rates per 1,00,000, Stratified by States in Adults aged ≥35 in the United States, 1999 to 2023** | |
| --- | --- |
| **State** | **Age-Adjusted Rate (95% CI)** |
| Alabama | 125.0 (123.3–126.6) |
| Alaska | 115.6 (110.4–120.9) |
| Arizona | 102.7 (101.5–103.9) |
| Arkansas | 135.7 (133.6–137.9) |
| California | 122.4 (121.8–123.0) |
| Colorado | 148.6 (146.8–150.5) |
| Connecticut | 101.0 (99.5–102.6) |
| Delaware | 137.7 (134.0–141.3) |
| District of Columbia | 109.4 (104.9–114.0) |
| Florida | 97.3 (96.7–97.9) |
| Georgia | 102.2 (101.1–103.3) |
| Hawaii | 88.9 (86.5–91.2) |
| Idaho | 155.0 (151.8–158.1) |
| Illinois | 105.1 (104.2–106.1) |
| Indiana | 157.1 (155.5–158.7) |
| Iowa | 137.0 (134.9–139.0) |
| Kansas | 124.7 (122.6–126.8) |
| Kentucky | 159.0 (157.0–161.0) |
| Louisiana | 115.2 (113.5–116.9) |
| Maine | 142.6 (139.6–145.6) |
| Maryland | 152.8 (151.2–154.5) |
| Massachusetts | 119.4 (118.0–120.7) |
| Michigan | 120.7 (119.7–121.8) |
| Minnesota | 159.1 (157.4–160.8) |
| Mississippi | 127.0 (124.8–129.2) |
| Missouri | 124.2 (122.8–125.6) |
| Montana | 124.5 (121.1–127.9) |
| Nebraska | 163.6 (160.6–166.6) |
| Nevada | 98.5 (96.5–100.6) |
| New Hampshire | 128.8 (125.8–131.8) |
| New Jersey | 110.1 (109.0–111.2) |
| New Mexico | 91.6 (89.5–93.7) |
| New York | 95.9 (95.3–96.6) |
| North Carolina | 135.4 (134.2–136.6) |
| North Dakota | 138.0 (133.7–142.3) |
| Ohio | 154.5 (153.4–155.6) |
| Oklahoma | 165.8 (163.5–167.9) |
| Oregon | 183.0 (180.8–185.1) |
| Pennsylvania | 137.2 (136.2–138.2) |
| Rhode Island | 136.7 (133.3–140.0) |
| South Carolina | 166.9 (165.0–168.8) |
| South Dakota | 143.9 (139.8–148.0) |
| Tennessee | 150.8 (149.3–152.4) |
| Texas | 134.7 (133.9–135.5) |
| Utah | 114.5 (112.0–116.9) |
| Vermont | 166.2 (161.3–171.0) |
| Virginia | 128.8 (127.4–130.0) |
| Washington | 154.2 (152.7–155.8) |
| West Virginia | 162.7 (159.8–165.5) |
| Wisconsin | 156.2 (154.5–157.8) |
| Wyoming | 151.5 (146.1–157.0) |

| **Supplemental Table 7: Arrhythmia–related Age-Adjusted Mortality Rates per 1,00,000, Stratified by Census Region in Adults aged ≥25 in the United States, 1999 to 2023** | | | | |
| --- | --- | --- | --- | --- |
| **Age-Adjusted Rate (95% CI)** | | | | |
| **Year** | **Northeast** | **Midwest** | **South** | **West** |
| **1999** | 111.1 (109.9–112.3) | 122.1 (120.9–123.3) | 108.3 (107.4–109.2) | 104.5 (103.3–105.7) |
| **2000** | 105.6 (104.5–106.8) | 115.8 (114.7–117.0) | 100.9 (100.0–101.8) | 100.3 (99.1–101.5) |
| **2001** | 109.5 (108.3–110.6) | 118.0 (116.9–119.2) | 106.8 (105.9–107.8) | 106.2 (105.0–107.4) |
| **2002** | 107.3 (106.1–108.4) | 120.0 (118.8–121.1) | 107.1 (106.2–108.0) | 107.1 (105.9–108.3) |
| **2003** | 104.1 (103.0–105.3) | 115.5 (114.4–116.6) | 105.4 (104.5–106.3) | 106.1 (104.9–107.3) |
| **2004** | 100.0 (98.9–101.1) | 111.8 (110.7–112.9) | 99.0 (98.1–99.8) | 102.0 (100.8–103.1) |
| **2005** | 100.9 (99.8–102.0) | 115.8 (114.7–116.9) | 104.1 (103.2–104.9) | 104.1 (102.9–105.2) |
| **2006** | 96.1 (95.0–97.1) | 112.8 (111.7–113.8) | 100.0 (99.2–100.9) | 102.1 (101.0–103.2) |
| **2007** | 95.4 (94.3–96.5) | 110.4 (109.3–111.5) | 98.7 (97.9–99.5) | 100.2 (99.1–101.2) |
| **2008** | 96.8 (95.8–97.9) | 112.3 (111.2–113.3) | 98.4 (97.6–99.3) | 101.4 (100.3–102.4) |
| **2009** | 93.5 (92.5–94.5) | 108.9 (107.8–109.9) | 95.5 (94.7–96.3) | 98.4 (97.4–99.5) |
| **2010** | 97.6 (96.6–98.7) | 108.8 (107.8–109.9) | 98.3 (97.5–99.1) | 101.7 (100.6–102.7) |
| **2011** | 99.9 (98.9–101.0) | 112.5 (111.4–113.5) | 98.8 (98.0–99.6) | 105.6 (104.5–106.6) |
| **2012** | 99.9 (98.8–100.9) | 113.0 (111.9–114.0) | 101.4 (100.6–102.2) | 105.6 (104.6–106.6) |
| **2013** | 102.5 (101.4–103.6) | 116.0 (115.0–117.1) | 104.0 (103.2–104.8) | 108.3 (107.3–109.3) |
| **2014** | 102.4 (101.3–103.4) | 116.7 (115.6–117.7) | 104.7 (103.9–105.5) | 108.0 (106.9–109.0) |
| **2015** | 104.6 (103.6–105.7) | 121.7 (120.7–122.8) | 108.6 (107.8–109.4) | 113.3 (112.3–114.4) |
| **2016** | 103.6 (102.6–104.6) | 120.9 (119.8–121.9) | 109.8 (109.0–110.6) | 115.4 (114.4–116.5) |
| **2017** | 105.7 (104.6–106.7) | 126.6 (125.5–127.6) | 113.7 (112.9–114.5) | 119.0 (118.0–120.0) |
| **2018** | 106.6 (105.6–107.7) | 129.6 (128.6–130.7) | 116.1 (115.3–116.9) | 118.6 (117.5–119.6) |
| **2019** | 107.3 (106.3–108.3) | 131.3 (130.2–132.3) | 118.9 (118.1–119.7) | 119.8 (118.8–120.8) |
| **2020** | 126.7 (125.6–127.8) | 152.6 (151.4–153.7) | 137.1 (136.3–137.9) | 133.7 (132.6–134.7) |
| **2021** | 131.6 (130.4–132.7) | 164.0 (162.7–165.2) | 155.3 (154.4–156.2) | 152.3 (151.1–153.4) |
| **2022** | 125.7 (124.6–126.8) | 153.0 (151.8–154.1) | 146.4 (145.5–147.2) | 144.0 (143.0–145.1) |
| **2023** | 120.5 (119.4–121.6) | 144.6 (143.5–145.7) | 141.4 (140.6–142.3) | 137.5 (136.4–138.5) |
| **Total** | **106.2 (105.1–107.3)** | **123.0 (121.9–124.1)** | **111.1 (110.3–112.0)** | **112.6 (111.5–113.7)** |

| **Supplemental Table 8 Arrhythmia-related Age-Adjusted Mortality Rates per 1,00,000, Stratified by Urban-Rural Classification in Adults aged ≥35 in the United States, 1999 to 2023** | | |
| --- | --- | --- |
| **Age-Adjusted Rate (95% CI)** | | |
| **Year** | **Metropolitan** | **Nonmetropolitan** |
| 1999 | 109.9 (109.3–110.6) | 118.1 (116.8–119.4) |
| 2000 | 103.8 (103.2–104.4) | 112.2 (111.0–113.5) |
| 2001 | 108.3 (107.7–108.9) | 117.0 (115.7–118.3) |
| 2002 | 108.4 (107.8–109.0) | 118.5 (117.2–119.8) |
| 2003 | 105.6 (105.0–106.2) | 116.8 (115.5–118.1) |
| 2004 | 100.8 (100.2–101.4) | 112.2 (110.9–113.4) |
| 2005 | 104.1 (103.5–104.6) | 115.9 (114.6–117.2) |
| 2006 | 100.5 (100.0–101.1) | 112.4 (111.1–113.6) |
| 2007 | 98.9 (98.3–99.4) | 111.2 (110.0–112.4) |
| 2008 | 99.5 (98.9–100.0) | 113.6 (112.4–114.8) |
| 2009 | 96.3 (95.8–96.8) | 110.7 (109.5–111.9) |
| 2010 | 98.7 (98.2–99.2) | 113.8 (112.6–115.0) |
| 2011 | 101.1 (100.6–101.7) | 115.7 (114.4–116.9) |
| 2012 | 101.8 (101.3–102.3) | 118.8 (117.6–120.1) |
| 2013 | 104.6 (104.1–105.1) | 121.4 (120.1–122.6) |
| 2014 | 104.3 (103.8–104.8) | 124.4 (123.2–125.6) |
| 2015 | 108.2 (107.6–108.7) | 130.5 (129.2–131.8) |
| 2016 | 108.8 (108.3–109.3) | 130.4 (129.1–131.6) |
| 2017 | 112.4 (111.9–112.9) | 135.8 (134.6–137.1) |
| 2018 | 113.2 (112.7–113.7) | 141.7 (140.4–143.0) |
| 2019 | 114.6 (114.1–115.1) | 145.2 (144.0–146.5) |
| 2020 | 132.2 (131.6–132.7) | 166.9 (165.5–168.3) |
| **Total** | **106.8 (106.7-106.9)** | **123.8 (123.5-124.1)** |


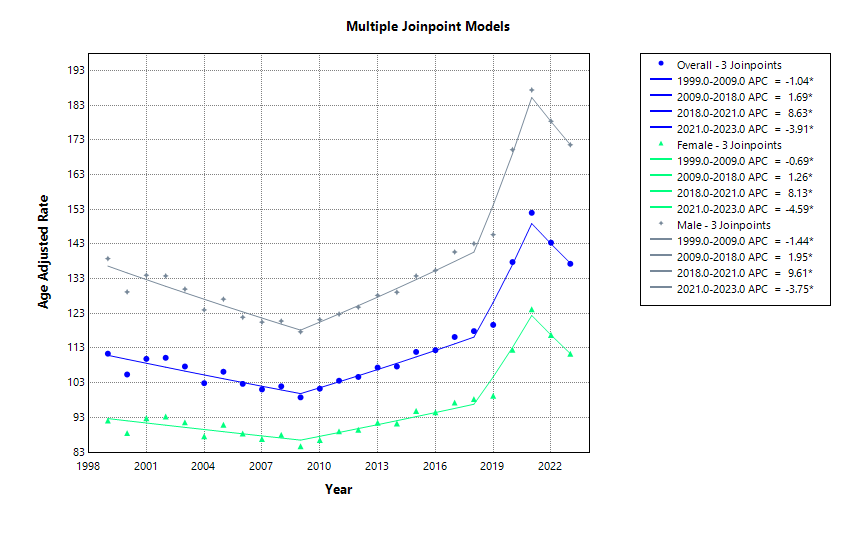


* Indicates that the APC significantly differs from zero at α = 0.05.

**Supplemental Figure 1**: Overall and Sex-Stratified arrhythmia-related AAMRs per 100,000 Among the population of the United States, 1999 to 2023.

**Supplemental Figure 2A:** Sensitivity analysis of arrhythmia-related AAMRs per 100,000.


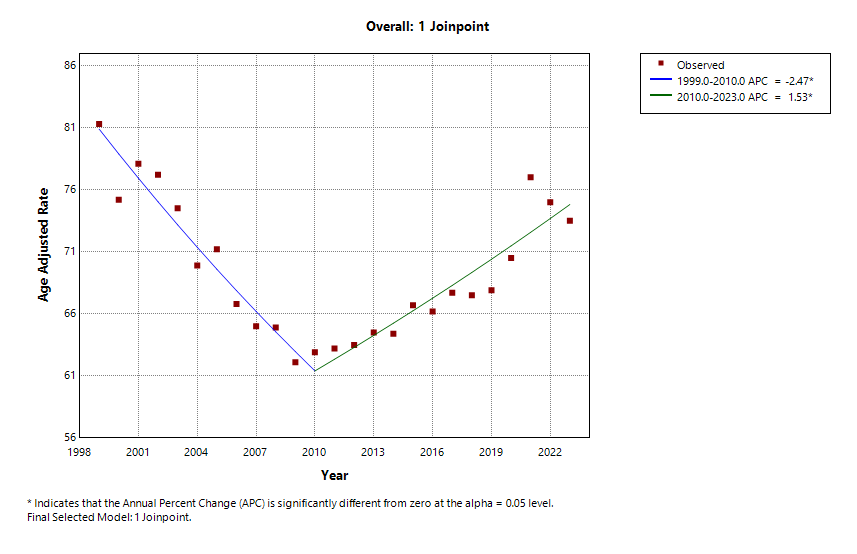


**Supplemental Figure 2B:** Jointpoint figure of sensitivity analysis of arrhythmia-related AAMRs per 100,000.


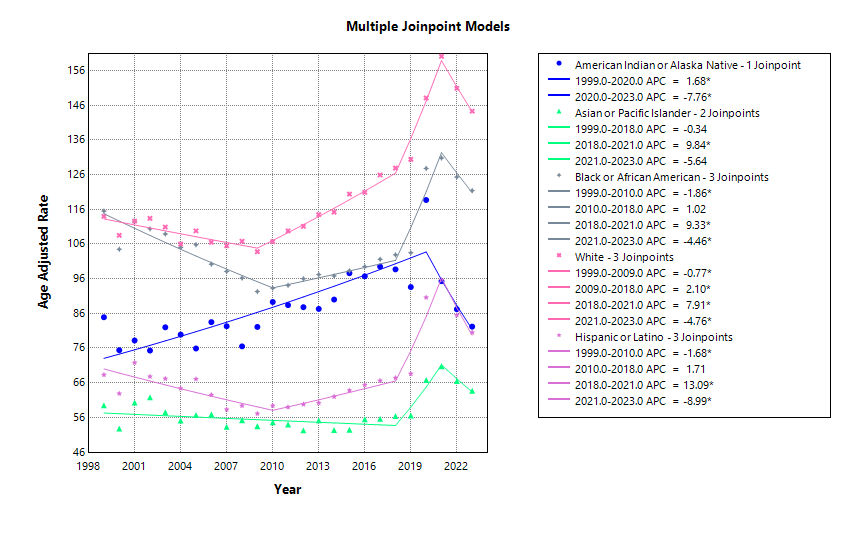


* Indicates that the APC significantly differs from zero at α = 0.05.

**Supplemental Figure 3:** Arrhythmia-related AAMRs per 100,000 Stratified by Race in the United States, 1999 to 2023.


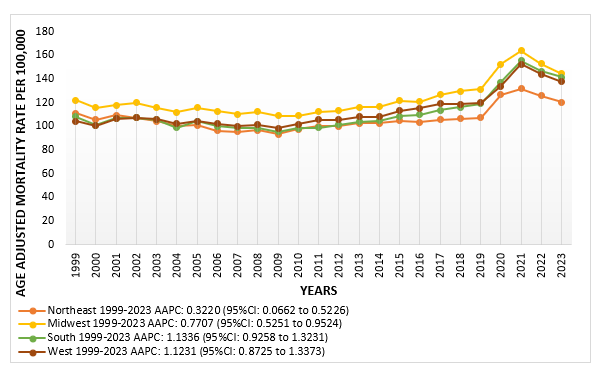


**Supplemental Figure 4:** Arrhythmia-Related AAMRs per 100,000 Stratified by Census Region in the United States, 1999 to 2023


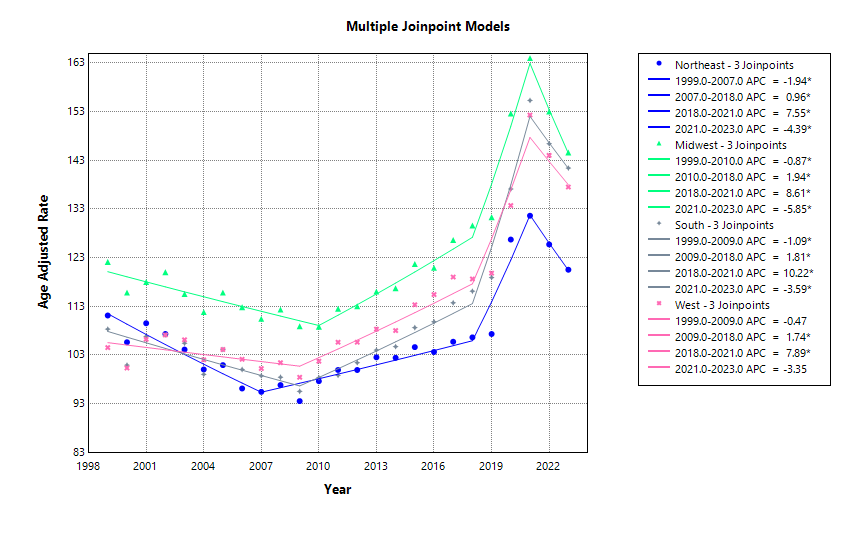


* Indicates that the APC significantly differs from zero at α = 0.05.

**Supplemental Figure 5:** Arrhythmia-Related AAMRs per 100,000 Stratified by Census Region in the United States, 1999 to 2023.


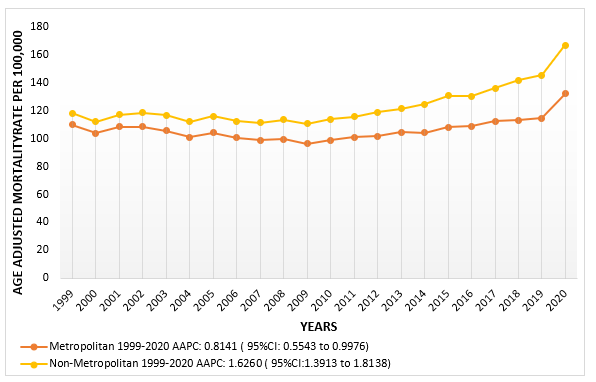


**Supplemental Figure 6:** Arrhythmia-Related AAMRs per 100,000 Stratified by Urbanization Among Older Adults in the United States, 1999 to 2023


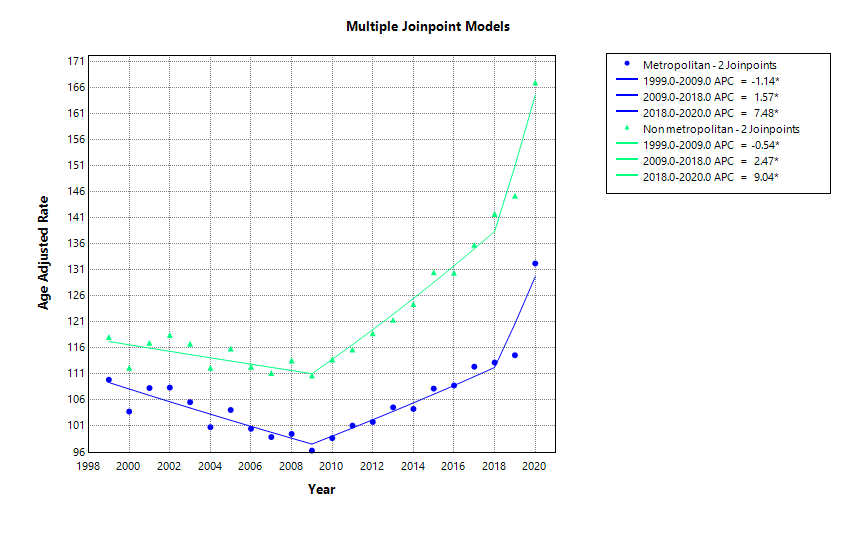


* Indicates that the APC significantly differs from zero at α = 0.05.

**Supplemental Figure 7:** Arrhythmia-Related AAMRs per 100,000 Stratified by Urbanization Among Older Adults in the United States, 1999 to 2023.

**Supplemental Figure 8:** Comparison of Arrhythmia and CVD-related Deaths
